# Supplementary material for: The Latent Aging of Cells
Source: bioRxiv. 2024 May 30:2024.05.28.596284. Preprint. [Version 1] doi: 10.1101/2024.05.28.596284 (PMC11160607; doi:10.1101/2024.05.28.596284)
Supplement: Supplement 2 [file NIHPP2024.05.28.596284v1-supplement-2.pdf]

bioRxiv preprint doi: https://doi.org/10.1101/2024.05.28.596284; this version posted May 30, 2024. The copyright holder for this preprint (which was not certified by peer review) is the author/funder, who has granted bioRxiv a license to display the preprint in perpetuity. It is made available under aCC-BY 4.0 International license.

Supplementary Figure 1: Density of DNAm clock CpGs in ELDAR's loadings

bioRxiv preprint doi: https://doi.org/10.1101/2024.05.28.596284; this version posted May 30, 2024. The copyright holder for this preprint (which was not certified by peer review) is the author/funder, who has granted bioRxiv a license to display the preprint in perpetuity. It is made available under aCC-BY 4.0 International license.

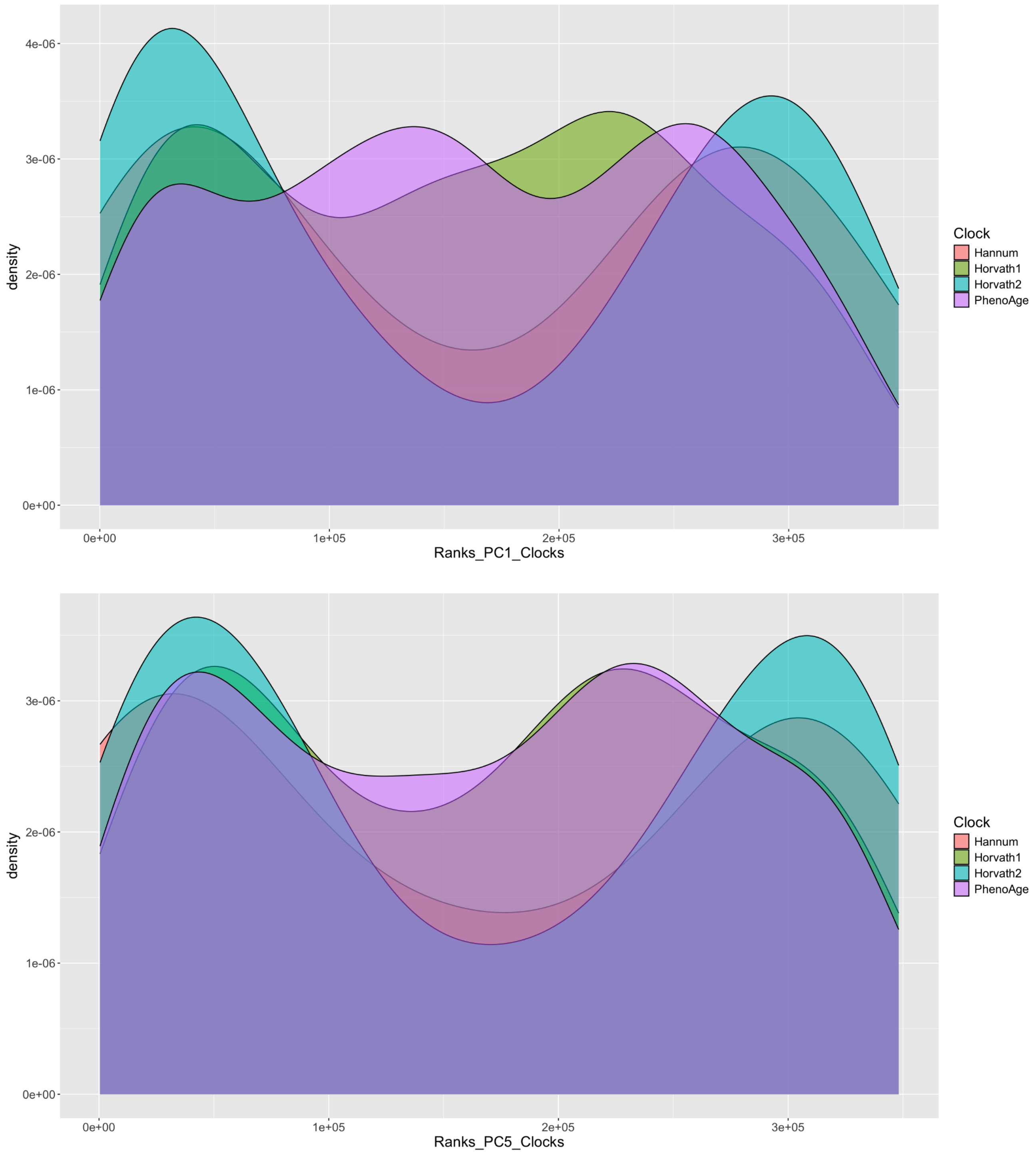

The Horvath2 and Hannum clocks have a correlation with the PC1 and PC5 measures presented here, while there is a negligible correlation with the Horvath1 and PhenoAge clocks. This was determined by arranging all the CpGs in the data by their PC loading and

bioRxiv preprint doi: https://doi.org/10.1101/2024.05.28.596284; this version posted May 30, 2024. The copyright holder for this preprint (which was not certified by peer review) is the author/funder, who has granted bioRxiv a license to display the preprint in perpetuity. It is made available under aCC-BY 4.0 International license.

comparing them to the clock CpGs. This supports the overlap found between the arbitrary cutoff of the top and bottom 1000 CpGs with the Hannum, Horvath Skin-Blood, Horvath Pan-Tissue, and PhenoAge clocks.
